# Identification of Neuronal Pentraxins as Synaptic Binding Partners of C1q and the Involvement of NP1 in Synaptic Pruning in Adult Mice

**DOI:** 10.3389/fimmu.2020.599771

**Published:** 2021-02-08

**Authors:** Réka Á. Kovács, Henrietta Vadászi, Éva Bulyáki, György Török, Vilmos Tóth, Dominik Mátyás, Judit Kun, Éva Hunyadi-Gulyás, Flóra Zsófia Fedor, Ádám Csincsi, Katalin Medzihradszky, László Homolya, Gábor Juhász, Katalin A. Kékesi, Mihály Józsi, Balázs A. Györffy, József Kardos

**Affiliations:** ^1^ ELTE NAP Neuroimmunology Research Group, Department of Biochemistry, Institute of Biology, ELTE Eötvös Loránd University, Budapest, Hungary; ^2^ Molecular Cell Biology Research Group, Institute of Enzymology, Research Center for Natural Sciences, Hungarian Academy of Sciences Centre of Excellence, Budapest, Hungary; ^3^ Department of Biophysics and Radiation Biology, Semmelweis University, Budapest, Hungary; ^4^ Laboratory of Proteomics, Institute of Biology, ELTE Eötvös Loránd University, Budapest, Hungary; ^5^ Laboratory of Proteomics Research, Biological Research Centre, Eötvös Loránd Research Network (ELKH), Szeged, Hungary; ^6^ Doctoral School of Chemical Engineering and Material Sciences, Pannon University, Veszprém, Hungary; ^7^ Complement Research Group, Department of Immunology, ELTE Eötvös Loránd University, Budapest, Hungary; ^8^ Department of Physiology and Neurobiology, Institute of Biology, ELTE Eötvös Loránd University, Budapest, Hungary

**Keywords:** synaptic pruning, neuronal pentraxin, complement component C1q, synaptosome, flow cytometry, immunostaining, complement classical pathway, microglial phagocytosis

## Abstract

Elements of the immune system particularly that of innate immunity, play important roles beyond their traditional tasks in host defense, including manifold roles in the nervous system. Complement-mediated synaptic pruning is essential in the developing and healthy functioning brain and becomes aberrant in neurodegenerative disorders. C1q, component of the classical complement pathway, plays a central role in tagging synapses for elimination; however, the underlying molecular mechanisms and interaction partners are mostly unknown. Neuronal pentraxins (NPs) are involved in synapse formation and plasticity, moreover, NP1 contributes to cell death and neurodegeneration under adverse conditions. Here, we investigated the potential interaction between C1q and NPs, and its role in microglial phagocytosis of synapses in adult mice. We verified *in vitro* that NPs interact with C1q, as well as activate the complement system. Flow cytometry, immunostaining and co-immunoprecipitation showed that synapse-bound C1q colocalizes and interacts with NPs. High-resolution confocal microscopy revealed that microglia-surrounded C1q-tagged synapses are NP1 positive. We have also observed the synaptic occurrence of C4 suggesting that activation of the classical pathway cannot be ruled out in synaptic plasticity in healthy adult animals. In summary, our results indicate that NPs play a regulatory role in the synaptic function of C1q. Whether this role can be intensified upon pathological conditions, such as in Alzheimer’s disease, is to be disclosed.

## Introduction

Complement components and pentraxins represent major humoral factors of the innate immune system, that often in collaboration play important roles in host defense, removal of dead cells, and several other physiological processes (1–4). In addition to acting systemically, complement and pentraxins are integral part of the neuroimmune axis and are involved in neurodegenerative diseases.

In the healthy brain, synaptic pruning is tightly regulated, primarily guided by an inherent developmental program and neuronal activity, persistently sculpting the global synaptic network suitable for optimal functioning. Maladaptive synaptic connectivity is usually a core component in the pathology of central nervous system (CNS) diseases, such as autism spectrum disorders (5) and schizophrenia (6). Moreover, excessive synapse loss is the hallmark of Alzheimer’s disease and other dementias (7, 8). A set of delicately regulated molecular machinery has evolved, having neuronal or non-neuronal origin, to tailor proper synaptogenesis and synapse elimination. Among them, a continuously growing body of evidence raises the role of the local complement system in synaptic pruning (9). Secreted microglial complement components C1q and C3b are deposited onto synapses to be eliminated leading to their recognition and engulfment by surrounding microglia (10, 11) in a yet not fully understood manner. Disturbances in complement-dependent synaptic pruning are in a causal relationship with the development of neurodegenerative disorders, schizophrenia, and epilepsy (5–8, 12), and recently reviewed by Tenner et al. (13), pointing out that properly functioning complement-mediated synapse elimination is indispensable for normal CNS functions. Even in adults, synapses are dynamically formed and eliminated as part of synaptic plasticity and linked to memory and learning. It is known that synaptic turnover can occur at high rates in adulthood (14). The average lifetime of synapses show large variety (15–17); synapses are constantly renewed, and thus it is relevant to carry out studies on adult mice.

Very little is currently known about the interaction partners of the initial complement component C1q in the CNS, especially on the synaptic surface that might drive synapse recognition. Earlier, we have shown in adult mice that C1q-tagging of synapses is linked to local apoptotic-like processes such as phosphatidylserine externalization (18), which has been recently pointed out as a key factor in developmental synaptic pruning by microglia (19). Nevertheless, members of the family of neuronal pentraxins (NPs) are suggested to be potential binding partners of C1q (20) because they share substantial structural homology with acute-phase immune proteins and well-known C1q binding partners: pentraxin 3 (PTX3) from the long-pentraxin family (21), the short-pentraxin C-reactive protein and serum amyloid P component (22). Intriguingly, mice lacking NP expression show defective synaptic refinement in the developing visual system (23), which is reminiscent of the deficits in input segregation described in C1q knock-out mice (10). NPs comprising NP1 and NP2, together with the neuronal pentraxin receptor (NPR) are abundantly present in the synaptic compartment and play a vital role in maintaining synaptic transmission and synaptic strength. Previous studies have established that NPs act *via* forming a large complex with each other and the AMPA-type glutamate receptors, thereby clustering on postsynaptic membranes of excitatory synapses (24, 25). Through their AMPA-receptor binding capabilities, NPs have emerged as potent regulators of excitatory synaptogenesis (26), functional synapse conversion (27), and synaptic plasticity (25, 28). Presynaptic release of NP2 from excitatory axon terminals is neuronal activity-dependent (29–31), and its secretion aids the homeostatic fine-tuning of synaptic transmission in local neuronal networks (32). On the other hand, it has been reported that NP1 acts in neuronal activity-independent manner, and its hetero-oligomers with NP2 exhibit higher clustering activity on AMPA-receptor than NP1 homo-oligomers (25). Apparently in contrast to its neuronal activity-independent synaptogenic roles, NP1 also contributes to neuronal cell death and neurodegeneration under adverse conditions in an activation dependent manner. During low neuronal activity, increased NP1 expression is evoked, triggering apoptotic neuronal cell death (33) mediated by glycogen synthase kinase-3 (Gsk3) activity (34), and involved in the mitochondrial accumulation of the pro-apoptotic BAX (35). Similarly, NP1 promotes neuronal cell death under hypoxic–ischemic conditions (36) and might be an important player in the pathomechanism of neurotoxic amyloid-beta-induced synapse loss, neurite damage, and cell death (37).

In sum, NPs are involved in synapse formation and plasticity; moreover, NP1 also serves as a neuronal mediator of harmful external stimuli directing affected neurons to apoptosis. In spite of the huge impact of both the complement and NPs on the synaptic network and their assumed binding capabilities to each other, it remained elusive whether their synaptic functions converge. Therefore, in this study, we systematically examined the potential interaction between C1q and NPs, particularly in the synaptic compartment in adult, wild-type mice. Moreover, we studied whether their interaction plays a role in the microglial phagocytosis of C1q-tagged synapses.

## Materials and Methods

### Animals

The experiments were conducted on in-house bred 6–8 months old male C57BL/6N mice. Animals were housed under standard laboratory conditions (12:12 h light–dark cycle with free access to water and food).

### Antibodies Used in This Study

AB1: anti-mouse synaptophysin (#101 006, Synaptic Systems, Göttingen, Germany); AB2: anti-mouse cytochrome c oxidase subunit 4 (Cox4, #sc-58348, Santa Cruz Biotechnology, Dallas, TX, USA (Santa Cruz)); AB3: anti-mouse actin-*β* (#AC026, Abclonal, Woburn, MA, USA); AB4: anti-mouse postsynaptic density protein 95 (Psd95, #MA1-045, Thermo Fisher Scientific (Thermo)); AB5: anti-mouse L-lactate dehydrogenase B (Ldhb, #PAB69Mu01, Cloude-Clone Corp.; Katy, TX, USA), AB6: anti-human and mouse neuronal pentraxin 1 (#20656-1-AP, Proteintech, Rosemont, IL, USA); AB7: anti-human and mouse neuronal pentraxin 2 (#sc-166035, Santa Cruz); AB8: anti-rabbit Alexa Fluor 488-conjugated (#711-545-152, Jackson ImmunoResearch Laboratories, West Grove, PA, USA (Jackson)); AB9: anti-mouse Alexa Fluor 647-conjugated (#715-605-151, Jackson); AB10: anti-human C1qb antibody (#H00000713-D01P, Abnova, Taipei, Taiwan); AB11: anti-mouse secondary antibody, HRP-conjugated (#715-035-150, Jackson), AB12: anti-His-tag antibody (#MA1-21315, Thermo), AB13: anti-human C4BP antibody (#MCA2609, Bio-Rad); AB14: anti-human C4 (A305, Quidel, San Diego, CA, USA); AB15: anti-goat HRP-conjugated (#P0449, Dako, Agilent, Santa Clara, CA, USA); AB16: anti-human neuronal pentraxin 1 (#STJ73037; St John’s Laboratory, London, UK); AB17: anti-human and mouse C1qA (#PAD207Mu01; Cloud-Clone Corp.); AB18: anti-goat Alexa Fluor 488-conjugated (#705-545-147; Jackson); AB19: anti-mouse Alexa Fluor 488-conjugated (#715-545-151, Jackson); AB20: anti-rabbit Alexa Fluor 647-conjugated (1:500 dilution; catalogue number: 715-605-151; Jackson), AB21: anti-mouse C1qA (# AB172451; Abcam, Cambridge, UK); AB22: anti-mouse Iba1 (#234 004, Synaptic Systems); AB23: anti-mouse Alexa Fluor 594-conjugated (#715-585-150, Jackson); AB24: anti-rabbit Cy3-conjugated (#711-165-152; Jackson); AB25: anti-chicken Cy5-conjugated (#703-175-155; Jackson); AB26: anti-guinea pig Alexa Fluor 488-conjugated (#706-545-148; Jackson); AB27: anti-human and mouse neuronal pentraxin 2 (# 10889-1-AP; Proteintech); AB28: anti-mouse synaptophysin (#ab8049; Abcam); AB29: anti-mouse MAP2 (# ab5392; Abcam); AB30: anti-chicken Cy3-conjugated (#703-165-155; Jackson); AB31: rabbit isotype control (#02-6102, Thermo); AB32: mouse isotype control (Mopc, BioLegend); AB33: anti-C4 (#PAA888Mu01, Cloud-Clone Corp.). Dilutions are shown in the descriptions of the corresponding methods.

### Primary Neuronal Culture

Primary neuronal cell culture was prepared from the cerebral cortices of mice on embryonic days 17–18 (E17–18). Subsequent to the euthanasia of the mother *via* cervical dislocation, E17–18 embryos were removed, and their cerebral cortices were collected free from meninges under aseptic conditions according to Mórotz et al. (38). After culturing the primary neurons for 14 days *in vitro*, the cell culture was subjected to immunofluorescence labeling (see the protocol below).

### Isolated and Recombinant Proteins

Recombinant human His-tagged NP1 and NP2 proteins were purchased from R&D Systems (Minneapolis, MN, USA; catalog numbers: 7707-NP-050 and 7816-NP-050). Factor H (FH) (catalog number: 341274) and human C1 (catalog number: 204873) were purchase from Merck Millipore (Billerica, MA, USA). The C1q was isolated from mouse serum in-house according to Györffy et al. (18), and His-tagged human PTX3 was prepared in-house (see below).

### Western Blotting

Protein samples were completed with an equal volume of reducing 2× Laemmli buffer, incubated at 96 °C for 5 min, and subjected to SDS-PAGE using 10% (w/v) polyacrylamide gels in all experiments. After separation, proteins were blotted to PVDF membranes at 100 mA for 1 h and then blocked with 5% (w/v) bovine serum albumin (BSA) in 0.1% (v/v) Tween-20, Tris-buffered saline (TBS-T) for 1 h at room temperature (RT). Membranes were incubated in blocking buffer overnight at 4 °C with primary antibodies as follows: either anti-Syp (AB1, 1:500), anti-Cox4 (AB2, 1:500), anti-actin-*β* (AB3, 1:10,000), anti-Psd95 (AB4, 1:1500), anti-Ldhb (AB5, 1:1,000), anti-NP1 (AB6, 1:1,000), or anti-NP2 (AB7, 1:1,000). Subsequently, blots were incubated in TBS-T for 2 h at RT with the proper secondary antibodies: either anti-rabbit Alexa Fluor 488-conjugated (AB8, 1:1,000) or, anti-mouse Alexa Fluor 647-conjugated (AB9, 1:1,000) antibody. Fluorescence of protein bands was detected with a Typhoon Trio+ scanner (Amersham Biosciences, Little Chalfont, UK).

### Plasmid, Transfection, and Cell Culture

The entire coding sequence of the human PTX3 cDNA (1,146 bp) was cloned into the BamHI and EcoRI sites of pPTX plasmid (modified pcDNA3.1+). The PTX3 construct contained His_6_-tag at the C terminus.

HEK-293 H cells, a kind gift of Árpád Mike (Eötvös Loránd University, Budapest, Hungary), were grown in ProCHO5 medium at 37 °C in a humidified incubator with 5% CO_2_/95% air. To generate a stable cell line, we transfected cells with a plasmid containing the human pentraxin 3 gene using Lipofectamine 3000 (Thermo Fisher Scientific, Waltham, MA, USA). Typically, 7 μg of DNA and 10 μl of Lipofectamine 3000 were used per T 25 cell culture flask. Geneticin (catalog number: 10131027, Thermo Fisher Scientific) was used as a selection marker. We started at 200 μg/ml protein concentration, and every second or third day, we duplicated the dose up to 1 mg/ml. PTX3 expression of the heterologous stable cell line was detected by Western blotting. Restriction and DNA modification enzymes, DNA and protein molecular weight standards were purchased from Thermo Fisher Scientific (Waltham, MA, USA).

### Nickel Affinity and Anion Exchange Chromatography

PTX3 was purified by Ni^2+^-affinity chromatography (Profinity IMAC Resin, Bio-Rad, Hercules, CA, USA). The column was equilibrated with a buffer containing 20 mM Tris, 300 mM NaCl, and 10 mM imidazole, pH 8. After incubating the cell lysate on the column, PTX3 was eluted using the same buffer supplemented with 500 mM imidazole. After dialyzing PTX3-containing fractions against 20 mM Tris pH 8 buffer containing 300 mM NaCl, samples were loaded on anion-exchange column (HiTrap Q HP, GE Healthcare, Chicago, IL, USA) equilibrated with the same Tris buffer. The protein was eluted by a linear NaCl gradient (300–1000 mM). In each purification step, the PTX3 content of the fractions was tested by Western blotting.

### Microtiter Plate Binding Assays

Costar microtiter plates (Corning, Corning, NY, USA) were coated with either NP1, NP2, or PTX3 (10 µg/ml) in phosphate-buffered saline (PBS) overnight at 4 °C. After blocking with 4% (w/v) BSA in 0.05% (v/v) Tween-20, PBS (PBS-T) for 1 h at RT, wells were incubated with C1q (5.6–45 µg/ml) diluted in PBS for 1 h at RT. The wells were washed with PBS-T and bound C1q was recognized *via* incubation with anti-C1qb antibody (AB10, 1:1000) in blocking solution for 1 h at RT. After washing with PBS-T, wells were incubated with HRP-conjugated anti-mouse secondary antibody (AB11, 1:8,000) in PBS-T for 45 min at RT. 3,3′,5,5′-tetramethylbenzidine (TMB) substrate (catalog number: 34028, Thermo Fisher Scientific) level was measured at 450 nm. To investigate whether pentraxins can bind to the C1 complex, FH, or C4b-binding protein (C4BP), microtiter plate binding assays were performed essentially as described above. Wells were coated with either C1 (5 µg/ml) or FH (10 μg/ml) and, after the blocking, incubated with neuronal pentraxins. For C1, 3.7–30 µg/ml NP1 and NP2, and 1.8–15 µg/ml PTX3 were used and for FH, 2.5–20 µg/ml NP1 and NP2 and 1.2–10 µg/ml PTX3 were used. To detect bound pentraxins, wells were incubated with an anti-His-tag antibody (AB12, 1:1,000) in blocking solution for 1 h at RT, and the signal was detected as described above. For C4BP, equal concentrations (10 µg/ml) of NP1, NP2, and PTX3 were coated and were blocked with 4% (w/v) BSA in PBS-T for 1 h at RT. Then wells were incubated with 6.2–100 µg/ml C4BP. To detect bound C4BPs, wells were incubated with an anti-C4BP antibody (AB13, 1:1000) in blocking solution for 1 h at RT, and the signal was detected as described above.

### Complement Activation Assays

We investigated complement cascade activation by neuronal pentraxins *via* the classical pathway. Nunc microplates (Thermo Fisher Scientific) were coated with either NP1, NP2, or PTX3 (10 µg/ml) and blocked with 4% (w/v) BSA in PBS-T for 1 h at RT. Next, wells were incubated with 2% (v/v) normal or C1q-depleted human serum (catalog number: 234401, Sigma-Aldrich, Saint Louis, MO, USA) in PBS containing Ca^2+^/Mg^2+^ (catalog number: 14040-091, Thermo Fisher Scientific) supplemented either with 20 mM EDTA or EDTA-free for 30 min at 37 °C. Complement activation was detected by quantifying the deposited C4b using anti-C4 primary (AB14, 1:1,000) and anti-goat HRP-conjugated (AB15, 1:2,000) secondary antibody.

### Subcellular Fractionation

After urethane anesthesia, mice were transcardially perfused with ice-cold PBS to exclude potentially interfering effects of blood-derived C1q. Then mice were decapitated, and their brains were quickly removed. Fractionation of subcellular compartments was started immediately after brain removal. The fraction of cortical synaptosomes was prepared as described in Hahn et al., 2009 (39). Briefly, the cerebral cortex samples were mechanically homogenized in a sucrose-based, iso-osmotic medium. Subsequently, a discontinuous density gradient was prepared, an ultracentrifugation step was applied, and the highly pure fraction of synaptosomes was collected from the interface between the two layers with different densities [validated in Györffy et al. (18)]. Synaptosome samples were either fractionated further immediately to obtain sub-synaptic compartments or kept at 4 °C for up to 16 h for immunolabeling.

A sub-synaptic fractionation procedure was applied to the synaptosomes prepared from the whole brains of mice in order to separate the compartments of synaptic plasma membrane, synaptic cytoplasm, and synaptic mitochondria in the required amounts of downstream investigations. Our aim was to unveil the distribution of NP1 and NP2 proteins among these sub-synaptic compartments. To this end, we performed the protocol described in Bermejo et al., 2014 with minor modifications (40). In brief, isolated synaptosomes were ruptured by exposure to hypo-osmotic conditions for 30 min at 4 °C and mechanical homogenization with a glass tissue homogenizer to enable complete lysis of the synaptosomes. After an ultracentrifugation step (25,000 × *g*, 20 min), the supernatant was collected designated as the cytoplasmic compartment comprising the soluble protein content and the synaptic vesicle fraction of the synaptosomes, and devoid of plasma membrane-attached insoluble supramolecular complexes (*e.g.*, the post-synaptic density). The pellet was resuspended, layered on top of a discontinuous sucrose density gradient, and ultracentrifuged (150,000 × *g*, 2 h). The synaptic plasma membrane fraction was collected from the lowest interface, while the synaptic mitochondrial fraction was recovered from the pellet *via* solubilization in 2× Laemmli buffer. The synaptic plasma membrane was pelleted by ultracentrifugation (200,000 × *g*, 30 min) and solubilized in 2× Laemmli buffer as well. Finally, proteins of the synaptic cytoplasmic compartment were acetone-precipitated and solubilized in the same denaturing buffer. Fractions were stored at −80 °C until further use.

The purity of the sub-synaptic compartment fractions was evaluated using compartment-specific protein markers. We have chosen postsynaptic density protein 95 (Psd95) localized only in the insoluble, membrane-bound postsynaptic protein scaffold and showing marked enrichment in the synaptic plasma membrane fraction (40). L-lactate dehydrogenase B chain (Ldhb) and cytochrome c oxidase subunit 4 (Cox4) were used exclusively as cytoplasmic (41), and mitochondria-specific (42) proteins, respectively. In each fraction, Psd95, Ldhb, and Cox4 levels were quantified after Western blotting *via* densitometry, and the composition of every fraction was assessed based on their relative amount in the lysates. A system of linear equations was formulated where *x*, *y*, and *z* variables represented the synaptic plasma membrane, cytoplasm and mitochondrial content of each fraction, respectively, and experimentally detected NP fluorescence intensity levels were paired with the corresponding fractions. Solving the equations revealed the NP levels for each type of sub-synaptic compartment. Statistically significant differences in the levels of NPs between the different compartments were determined using one-way repeated measures ANOVA. In case of statistically significant difference between any of the investigated groups, the Bonferroni *post hoc* test was employed.

### Immunolabeling of Synaptosomes and Flow Cytometry

Immunolabeling of synaptosome fraction and analysis *via* flow cytometry were carried out as described previously (18) with minor modifications. In brief, synaptosomes were gently fixed with 0.25% (w/v) formaldehyde in sucrose-Tris buffer (320 mM sucrose, 5 mM Tris, pH 7.4). This iso-osmotic buffer possessing low ionic strength is fundamental for preventing synaptosome-aggregation (43). On the other hand, we omitted EDTA from the traditional sucrose-EDTA-Tris buffer (18, 43) in order to prevent deterioration of target-binding capabilities of C1q (44). After blocking aspecific interactions with 1% (w/v) BSA in sucrose-Tris buffer, the samples were incubated with either anti-neuronal pentraxin 1 (AB 16, 1:100) or anti-neuronal pentraxin 2 (AB7, 1:100), with or without anti-C1qA (AB17, 1:100) primary antibodies for 60 min at RT. After washing, either anti-goat Alexa Fluor 488-conjugated (AB18, 1:500) or anti-mouse Alexa Fluor 488-conjugated (AB19, 1:500) with or without anti-rabbit Alexa Fluor 647 (AB20, 1:500) secondary antibodies were applied for 45 min at RT to detect NP1, NP2, and C1qA, respectively. Finally, samples were extensively washed and then filtered through a 5.0-µm Durapore membrane filter (Merck Millipore).

Flow cytometry analysis of fluorescently immunolabeled synaptosomes was carried out as described earlier (18) using a BD FACSAria III sorter (BD Biosciences, San Jose, CA, USA) coupled with BD FACSDiva software (BD Biosciences). Gating was set the way it resulted 1% false positive for secondary control sample. For testing the viability of synaptosomes and validating the exclusive binding of antibodies to the synaptic surface, calcein-AM labeling (Thermo Fisher Scientific) was applied following the manufacturer’s instructions. Calcein labeling was done separately, and the calcein-labeled samples went through the same procedure as the antibody-labeled samples.

### Immunofluorescence Staining of Mouse Brain Sections

Urethane-anesthetized mice were transcardially perfused with ice-cold 0.1 M phosphate buffer (PB), pH 7.4, and subsequently, with 2% (w/v) formaldehyde, 0.1 M PB. After perfusion, the brains were removed and post-fixed for an additional 3 h using the same fixative solution at RT. After the washing steps with 0.1 M PB, 60 µm-thick, sagittal brain sections were produced using a vibratome, which was followed by additional extensive washing with 0.1 M PB. For the immunostaining, sections were washed first with TBS and then incubated in a blocking buffer (150 mM NaCl, 50 mM Tris, 100 mM L-lysine, 3% (w/v) BSA, pH 7.4) for 45 min at RT. Subsequently, brain sections were incubated in primary antibody buffer (150 mM NaCl, 50 mM Tris, 100 mM L-lysine, 1% (w/v) BSA, pH 7.4) containing either anti-neuronal pentraxin 1 (AB16, 1:100) or anti-neuronal pentraxin 2 (AB7, 1:50) together with anti-C1qA (AB21, 1:1,000), and anti-synaptophysin (AB1, 1:500) primary antibodies for 3 days at 4 °C. A group of brain sections was also labeled with anti-Iba1 (AB22, 1:200) primary antibody, as well. After washing with TBS, sections were incubated for 3 h at RT with either anti-goat Alexa Fluor 488-conjugated (AB18, 1:400), anti-mouse Alexa Fluor 594-conjugated (AB23, 1:400), together with anti-rabbit Cy3-conjugated (AB24, 1:400) and anti-chicken Cy5-conjugated (AB25, 1:400) secondary antibodies to detect NP1, NP2, C1q, and synaptophysin (Syp), respectively. For Iba1 (Ionized calcium-binding adapter molecule 1) detection, anti-guinea pig Alexa Fluor 488-conjugated (AB26, 1:400) secondary antibody was used. For C4 staining, brain sections were labeled with anti-C4 (AB33, 1:100) antibody and anti-synaptophysin (AB1, 1:500) primary antibodies for three days at 4 °C. Labeling with secondary antibodies was similar as above using anti-rabbit Alexa Fluor 488-conjugated (AB8, 1:400), together with anti-chicken Cy3-conjugated (AB30, 1:400) secondary antibodies to detect C4 and synaptophysin (Syp), respectively.

Immunostained sections were washed with TBS, mounted on glass slides using Aqua-Poly/Mount medium (catalog number: 18606-20; Polysciences, Warrington, PA, USA), and the slides were covered with cover glass (catalog number: CS2440100, Menzel Gläser, No. 1).

### Immunofluorescence Staining of Mouse Primary Cortical Neurons

The glass coverslips with primary neurons were incubated in PBS and subsequently, fixed with 4% (w/v) formaldehyde at RT for 15 min and washed with PBS three times. We incubated the coverslips in blocking buffer (PBS containing 0.3% (v/v) Triton X-100, 3% (w/v) BSA, pH 7.4) at RT for 1 h. Subsequently, coverslips were incubated in primary antibody buffer (PBS containing 0.3% (v/v) Triton X-100, 3% (w/v) BSA, pH 7.4) containing either anti-neuronal pentraxin 1 (AB16, 1:1,000) or anti-neuronal pentraxin 2 (AB27, 1:100) together with anti-synaptophysin (AB28, 1:100) and anti-MAP2 (AB29, 1:10,000) O/N at 4 °C. After extensive washing with PBS, coverslips were incubated for 1.5 h at RT with either anti-goat Alexa Fluor 488-conjugated (AB18, 1:800) or anti-rabbit Alexa Fluor 488-conjugated (AB8, 1:800), together with anti-chicken Cy3-conjugated (AB30, 1:800), and anti-mouse Alexa Fluor 647-conjugated (AB9, 1:800) secondary antibodies to detect NP1, NP2, MAP2 (microtubule-associated protein 2), and Syp, respectively. Immunostained coverslips were washed with PBS and mounted on glass slides using Aqua-Poly/Mount medium (catalog number: 18606-20; Polysciences).

### High-Resolution Confocal Microscopy and Image Analysis

Leica HyVolution 2 pseudo-super-resolution imaging was performed by a Leica TCS SP8 STED microscope using a Leica HC PL APO 100× STED white (1.4 NA) objective (Leica Microsystems, Wetzlar, Germany). The fluorescence of each dye was detected sequentially by using a hybrid detector and spectral detections. When three-labeled samples were imaged, the fluorescence of Alexa Fluor 488, Cy3, and Cy5 dyes were detected between 500–544 nm, 562–602 nm and 650–700 nm wavelengths at 488, 552, and 638 nm excitations, respectively. In the case of the samples with four labels, we used 488 nm (for Alexa Fluor 488), 552 nm (for Cy3 and Alexa Fluor 594) as well as 638 nm (for Alexa Fluor 647) wavelength lasers, and 504–546 nm, 589–614 nm, 630–652 nm, and 658–700 nm emission filters, respectively. Huygens Pro deconvolution software was used for image restoration and Leica LAS X 3.1.1 software was used for image analysis. The lateral dimensions of the acquired images were ~50 × 50 µm, whereas the axial dimension was ~3 µm. To make the investigation unbiased, the 50 × 50 µm fields were randomly selected in the neuropil of the approximately 1,500 × 1,500 µm large somatosensory area. Eighteen 3D-images were collected from nine sections of three animals; each image was stacked in 15–25 planes.

Colocalization analysis was carried out using an automated high-throughput workflow specifically tailored to aid the reliable identification of immunolabeled proteins and assessment of the degree of their colocalization. This approach is based on the Fiji image-analysis platform [an extended version of the ImageJ program (45)] and on a combination of its suitable plugins. The herein described protocol enables the colocalization analysis of C1q, NP1/2, and Syp proteins, all of which are distributed on the brain sections as discrete spots (diameters <~1 µm in the XY plane]. Images were split according to the fluorescence channels, and individual images were subjected to image segmentation *via* a two-step process. First, local maxima of individual spots (peaks of fluorescence intensity within a predefined radius in the X, Y, and Z planes) were identified using the 3D Maxima Finder plugin. Subsequently, segmentation of spots was carried out employing the 3D Spot Segmentation plugin. In brief, after applying a “watershed” process on the original images, individual spots were segmented using both the original and local maxima images based on the “local mean” thresholding method. The threshold values were set between 5,000 and 7,000 in the 16 bit (0–65,535) intensity scale. Pairwise colocalization analyses between C1q, NP1/2, and Syp proteins were carried out using their corresponding segmented images. Objects-based analyses were performed using the JACoP [“Just Another Colocalization Plugin” (46)], working on distances between objects’ geometrical centers (centroids). Briefly, an ellipsoid was calculated around every centroid with the maximal lateral and axial sizes of 200 nm and 500 nm, respectively (approximating the point spread function of the object). Particles were considered colocalizing if their corresponding ellipsoids were overlapping in the 3D space. After the pairwise analyses, evaluation of colocalization was also implemented between those C1q and NP1/2 centroids that simultaneously showed colocalization with Syp centroids, as well. The latter examination revealed the degree of colocalization between synaptic C1q and NP1/2 proteins. We also tested whether the close proximity of synaptic C1q and NP1/2 proteins in overall was solely accidental consequently to their high abundance in the synapse-rich cerebral cortex. For this, we utilized the Distance Analysis (DiAna) plugin (47) to randomly shuffle in the 3D space the centroids of one of the channels before the pairwise comparison of the two channels and the calculation of the cumulative frequency distribution of the minimal center-to-center distances for an image. The Monte Carlo simulation was automatically performed 100-times, and the mean cumulative frequency distribution and its 95% confidence interval envelope were calculated for the shuffled images. As the cumulative frequency distribution of the observed minimal center-to-center distances was not normally distributed, we compared the difference between the observed distributions with the mean shuffled distribution using a non-parametric statistical approach. Thus, we employed the Wilcoxon signed-rank test, and compared the median of the observed cumulative frequency distribution with that of the corresponding mean shuffled distribution.

C4, NP1, and Syp colocalization analysis was performed by using the same method as described above.

Finally, we assessed the degree of colocalization of NP1/2 with C1q-colocalizing Syp spots within the microglia to address whether C1q-tagged eliminated synapses preferentially contain NPs or not. This analysis involved the identification of C1q- and NP1/2-colocalizing Syp centroids using the scheme described above. Microglial cells were reconstructed using microglial Iba1 staining and pixel intensity threshold-based image segmentation in Fiji. 3D-rendered reconstruction of the same stack of images created only for visualization purposes, using the 3D Viewer plugin of Fiji, displayed using its “Surface” function with a threshold set to either 100 (for C1q, NP1/2, and Syp) or 50 (for Iba1). Subsequently, the number of those C1q-colocalized Syp centroids that were overlapping with the Iba1 staining with or without NP1/2 proteins were evaluated using JACoP plugin’s centers-particles coincidence function.

### Co-Immunoprecipitation

Co-immunoprecipitation experiments were performed to identify the direct or indirect interaction partners of synaptic NP1 and C1q. Cortical synaptosomes were lysed *via* mechanical homogenization with Sample Grinding Kit (catalog number: 80-6483-37, GE Healthcare), according to the manufacturer’s instructions, in TBS containing 1% (v/v) Triton X-100, 1 mM CaCl2, 1 mM MgCl2, supplemented with protease and phosphatase inhibitor cocktails (catalog numbers: P8340 and P5726, respectively, Sigma-Aldrich, Saint Louis, MO, USA) at 4 °C. Subsequently, lysates were pre-cleared with ProteinA/G Sepharose beads (catalog number: ab193262, Abcam) strictly following the manufacturer’s instructions, and incubated overnight at 4 °C with one of the primary antibodies as follows, depending on the experiment: anti-NP1 (AB6, 2 µg/sample), anti-C1qA (AB17, 2 µg/sample), rabbit isotype control (AB31, 2 µg/sample), mouse isotype control (AB32, 8 µg/sample). On the next day, samples were incubated with the beads for 1 h at 4 °C. Unbound proteins were completely removed after consecutive centrifugation steps, and then, captured proteins were eluted by incubating the beads at 96 °C for 5 min in 2× Laemmli buffer. The samples were run on SDS-PAGE, and the whole lanes were subjected to protein identification.

### Mass Spectrometry-Based Protein Identification

Protein identification by mass spectrometry was carried out as described previously (48). Briefly, each gel-lane was cut to five pieces, and the proteins were reduced, alkylated, and in-gel digested with trypsin (sequencing grade modified, side chain protected porcine trypsin, Promega, Madison, WI, USA) following the protocol by Shevchenko et al. (49). The HPLC-MS/MS analysis of the tryptic peptide mixtures was performed using a nanoflow UHPLC system (Waters M-Class UPLC) coupled to an LTQ-Orbitrap Elite (Thermo Fisher Scientific, Bremen, Germany) fitted with a nanospray ion source. 5 µl of tryptic peptides was injected into a trap column and then separated on a reverse-phase nano column. The elution of the peptides to the emitter tip was achieved using a flow rate of 250 nl/min and a 36 min long gradient increasing from 10 to 40% of solvent B (solvent A was water containing 0.1% formic acid, and solvent B was acetonitrile containing 0.1% formic acid).

## Results

### Neuronal Pentraxins Bind to C1q and C1 and Activate the Classical Complement Pathway

**Figure 1:**
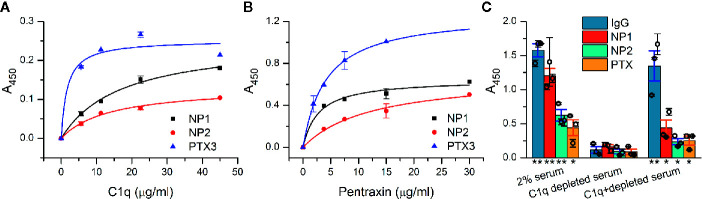
Interaction of neuronal pentraxins with C1q and C1 *in vitro* and activation of the complement classical pathway. **(A)** In an ELISA assay, NP1, NP2, and the positive control PTX3 were coated, and then, wells were incubated with purified C1q, and binding was detected with anti-C1q antibody. C1q showed direct interaction with both NP1 and NP2 in a dose-dependent manner, comparable to PTX3. Means ± SEM; *n* = 5. **(B)** Assay results with C1 complex immobilized. Recombinant NPs were detected with anti-His antibody and exhibited dose-dependent binding (representative experiment, Means ± SEM; *n* = 4.) Plots in **(A, B)** were fitted with hyperbolic function. **(C)** NP-dependent activation of the complement classical pathway was examined using microtiter plate binding assay. Neuronal pentraxins were able to activate the cascade in the presence of 2% serum, similarly to the positive controls (IgG and PTX3), as indicated by the deposition of C4 fragments, detected with anti-C4 antibody. In C1q-depleted serum, activation of the classical pathway by any of the investigated activators was dropped to baseline level. Supplementation of C1q-depleted 2% serum with purified C1q in the physiological concentration range (1.4 µg/ml) restored the activation. Columns represent the overall means ± SEM. Three biological replicates (each is a mean of two technical parallels) are also presented with hollow circles. The statistical analysis was two-sample t-test on normally distributed samples compared to the C1q-depleted respective samples (*p < 0.05, **p < 0.01, n = 3 biological replicates with two technical parallels).

The presumed interaction between NPs and C1q was first examined *in vitro* using recombinant proteins in a microtiter plate binding assay. NP1, NP2, and the positive control PTX3 were coated and then incubated with purified C1q. Confirming our initial assumption, C1q showed direct interaction with both NP1 and NP2 in a dose-dependent manner, comparable to the positive control PTX3 (**Figure 1A**). Our additional, reverse experiments showed the same binding characteristics between pentraxins and C1q when purified C1q was coated (**Supplementary Datasheet 2**). We addressed whether NPs could also bind to the entire C1 complex consisting of C1q and serine proteases C1r and C1s. Similar to the results above, dose-dependent binding of NPs and PTX3, as well, to C1 was observed using ELISA (**Figure 1B**). We further investigated if the demonstrated interactions are functional and strong enough to trigger activation of the classical pathway of the complement system. Thus, we coated NPs and conducted a complement activation assay using 2% normal and C1q-depleted human sera. According to Seelen et al., the 2% serum is sufficient to activate the classical pathway exclusively through C1q (50). Our results show that NPs are able to activate the classical pathway, measured as deposited C4 fragments in the presence of the C1 complex similar to the positive control Immunglobulin G (IgG) and PTX3. On the other hand, NPs, IgG, and PTX3 equally failed to activate the complement cascade in the absence of C1q from the serum indicating their contribution to complement activation *via* the classical pathway instead of the alternative or lectin routes (**Figure 1C**). Note that full recovery of complement activation was not observed using C1q-depleted serum with externally added C1q, in the assay utilizing neither the NPs, nor the positive control IgG and PTX3 proteins. Nevertheless, the added C1q substantially facilitated complement activation using all the investigated pentraxins and the IgG protein compared to the C1q-lacking condition.

### Neuronal Pentraxins Bind C4BP and Factor H

Several host ligands bind C1q and thus activate complement, but soluble complement regulatory proteins C4BP and factor H bound at the same time limit complement activation to the C3 level (51). PTX3 is known to bind C4BP, an inhibitor of the classical pathway (52). We investigated the interaction of NPs with C4BP and found a dose-dependent binding profile similar to that of PTX3 (**Supplementary Datasheet 2**). Factor H, an inhibitor of the alternative pathway also bound NPs and PTX3 when it was coated to ELISA plate (**Supplementary Datasheet 2**).

### NP1 Co-Immunoprecipitates With C1q in the Synaptic Lysate

After demonstrating the binding C1q to NPs *in vitro*, we investigated if the interaction could occur *in vivo* in the synaptic environment using co-immunoprecipitation followed by mass spectrometry-based protein identification. Using NP1-specific primary antibody, NP1 was pulled down together with NP2, all three chains of C1q (C1qA, C1qB, and C1qC), the pentraxin receptor (NPTXR), and in some samples, glutamate receptors Gria2 and Grm3. Co-immunoprecipitation using anti-C1qA antibody captured the other C1q chains C1qB and C1qC, while experiments using rabbit and mouse isotype control antibodies showed trace amounts of C1qB and C1qC chains (the results of mass spectrometry is attached as **Supplementary Datasheet 1**). Our results suggest that NP1 and C1q might be actual *in vivo* binding partners. The interaction of NP1 with NP2, NPTXR and the AMPA glutamate receptors detected in these experiments corroborates with earlier findings (24, 25).

### Neuronal Pentraxins Are Present in Synapses of the Primary Neurons

**Figure 2:**
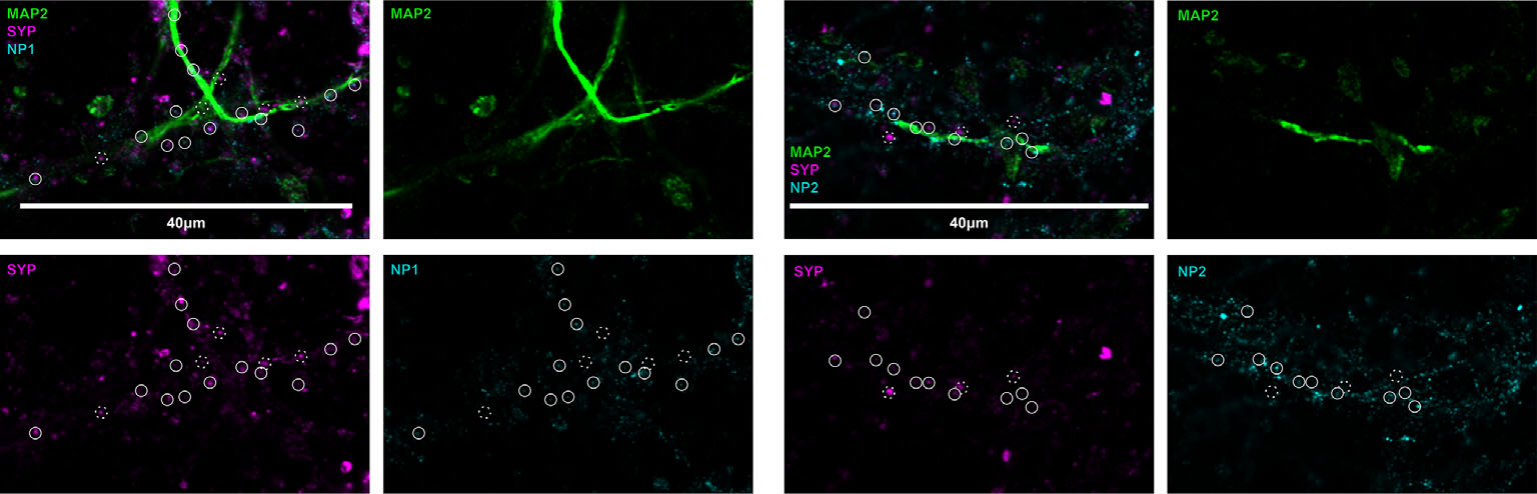
Representative image of neuronal pentraxins along the axons. Mouse primary cortical neurons were immunostained for MAP2, Syp, and either NP1 or NP2. On the representative images, synaptic NPs are clearly apparent, whereas synapses without NP-staining are also visible. Solid circles show synaptophysin signals colocalized with either NP1 or NP2, while dashed circles show synapses, which do not contain the neuronal pentraxins.

To visualize the location of neuronal pentraxins along the neurons, we immunostained mouse cerebral cortical primary neurons. Pyramidal cell dendrites were visualized with MAP2, while individual synapses with Syp labeling. Experiments identified both NP1 and NP2 colocalizing with synapses in the primary neuronal cell culture. On the other hand, the multiple immunostaining also revealed that some synapses are not NP-positive along the dendrite. The distribution of these proteins suggests a differential function of NPs (**Figure 2**). It is known that NP1 and NP2 are mainly or even exclusively localized to excitatory synapses (26–28, 53, 54).

### C1q Is Exclusively Present on the Surface of NP1/2 Positive Synaptosomes

**Figure 3:**
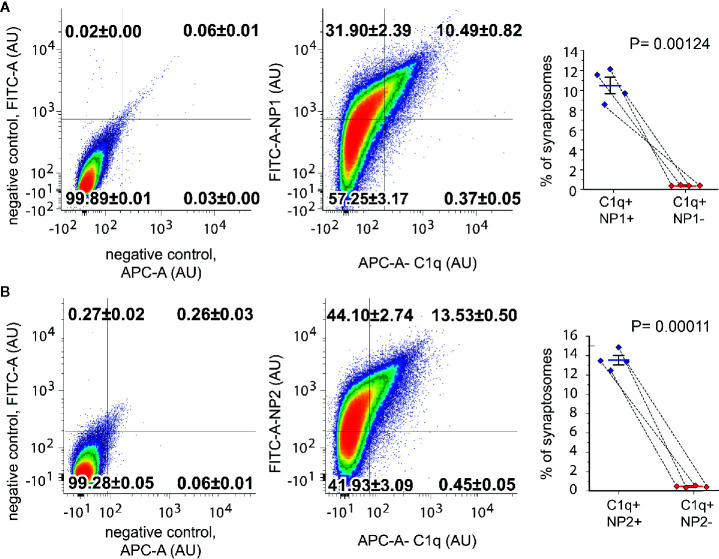
Co-occurrence of the C1q-tag with NP1/2 on synaptosomes examined by flow cytometry. According to the gating criteria, C1q-labeled synaptosomes were almost exclusively (~97%) positive for NP1 **(A)** and NP2 **(B)**. In contrast to C1q labeling, only ~24% of NP-labeled synaptosomes were positive for C1q. Density plots show representative measurements where the percentages of synaptosomes that belong to each quadrant were also indicated. The secondary antibody controls went through the same procedure as the fully labeled samples. Statistically significant differences were determined with two-tailed Student’s *t*-test of paired samples (Means ± SEM; *n* = 4 mice). To test the viability of synaptosomes, calcein-AM labeling was applied separately (**Supplementary Datasheet 2**).

To test whether C1q and NPs colocalize *in vivo* in the intact synapse, we performed double immunolabeling of isolated synaptosomes for C1q and NP1/2 and carried out their subsequent flow cytometry analysis. Flow cytometry was conducted by following our rigorously validated and currently published protocol (18). Our data demonstrated that 10.85 ± 0.81% (mean ± SEM) of synaptosomes were tagged with C1q, and ~97% of them (10.49 ± 0.82% of the recorded synaptosomes) were labeled with the anti-NP1 antibody as well (**Figure 3**). In contrast, out of the 42.38 ± 3.20% NP1 positive synaptosomes, only one fourth (~24%) were C1q-positive (10.49% ± 0.82% of the synaptosomes were double-positive). In case of NP2, multiparametric analysis indicated a similar labeling pattern: 13.9 ± 0.5% (mean ± SEM) of synaptosomes were tagged with C1q, and ~97% of them (13.53 ± 0.5% of the synaptosomes) were labeled with the anti-NP2 antibody, as well (**Figure 3**). Summarizing our data, nearly all of the C1q-tagged synaptosomes contained extracellular NP1 and NP2, further strengthening our hypothesis that they can interact *in vivo* on the synaptic surface. On the other hand, a considerably high portion of NP1/2-positive synaptosomes (~75%) appeared C1q-untagged that could be explained by C1q-independent extracellular functions of NPs. **Supplementary Datasheet 2** shows control measurements on non-labeled, only secondary antibody-labeled, only primary antibody-labeled samples, demonstrating that there was no spillover between the channels APC and FITC.

### Neuronal Pentraxin-1/2 Colocalize With C1q in the Synapses *In Vivo*

**Figure 4:**
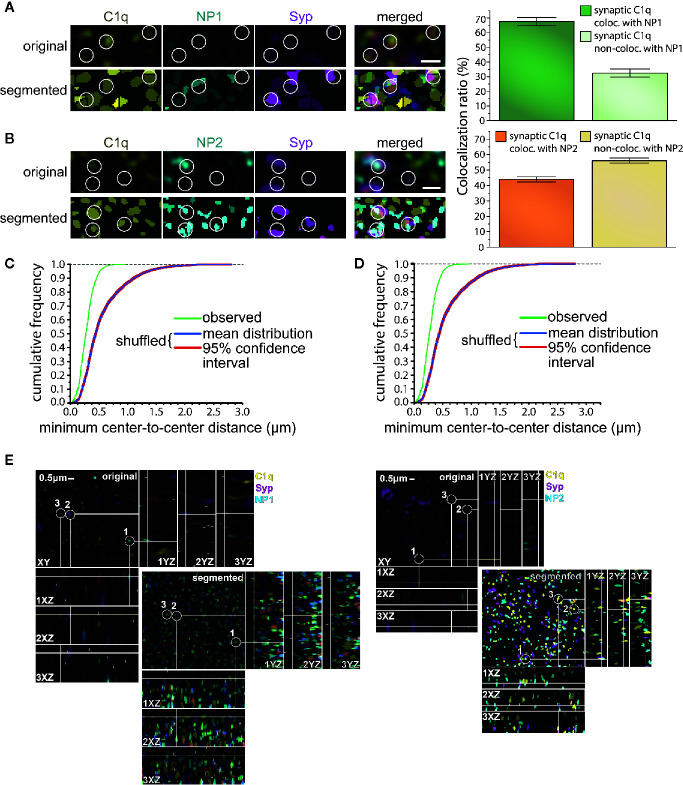
Colocalization of synaptic C1q with NPs in mouse brain sections. **(A, B)** High-resolution confocal microscopy images of triple immunostained cerebral cortical sections were segmented to identify individual C1q, NP1/2, and Syp spots. White circles indicate several triple-colocalizing spots automatically identified according to the predefined criterion. Analyses demonstrated extensive colocalization of synaptic C1q with synaptic NPs, particularly with NP1. (T-test results: (NP1) P = 2,19007E-10, (NP2) P = 4,71338E-06 on normally distributed samples.) **(C, D)** Close proximity of synaptic C1q with NP1 **(C)** and NP2 **(D)** is not the mere consequence of their high abundance. Medians of the minimal center-to-center distance’s cumulative frequency distributions significantly differ between the observed and randomly shuffled samples (*P* = 0.00019, Wilcoxon signed-rank test). Means ± SEM are shown; *n* = 18 3D-images recorded from sections of three mice. Scale bar = 0.5 µm. **(E)** The lateral view of the colocalized proteins.

The high degree of the simultaneous synaptic presence does not necessarily imply that C1q and NPs are present close enough to each other to assume their physical binding. Thus, our next objective was to verify their *in vivo* synaptic colocalization at higher resolution *via* immunostaining of mouse cortical brain sections combined with pseudo-super-resolution confocal microscopy and automated image analysis. This approach permitted us to systematically and reliably evaluate the colocalization of C1q and NPs with the synaptic marker Syp, and ultimately, the level of colocalization between Syp-colocalized (synaptic) C1q and NPs. The first step of the image analysis was segmentation of C1q, NP1/2, and Syp fluorescent signals that resulted in 3D spot-like objects. Subsequently, positions of the objects were computationally identified based on the exact location of their centroids. Ellipsoids having 200 nm and 500 nm maximal lateral and axial sizes, respectively, were automatically drawn around every centroid, and objects with overlapping ellipsoids were considered colocalizing. Image analysis revealed that ~67 and ~44% of synaptic C1q colocalize with synaptic NP1 and NP2, respectively (**Figure 4**), supporting our prior results on their direct interaction. Importantly, it seems unlikely that merely the high abundance of synaptic C1q and NPs in the neuropil accounts for their strong colocalization, as segmented C1q objects shuffled randomly failed to exhibit a similarly close proximity to NPs (**Figures 4C, D**). Comparing the two NP species, it is interesting to note that microscopy data implies that rather the synaptic NP1 but not NP2 could be the preferable target of C1q. Although we observed a particularly high degree of synaptic colocalization between C1q and NPs with this approach, the results fall short compared to the flow cytometry data showing externally located NPs on ~97% of C1q-positive synaptosomes. This result may support the idea that not all the NPs detected on synaptosomal surfaces were C1q-bound because they possess C1q-independent functions (24, 25, 32, 55). In addition, flow cytometry analysis presumably showed a bias towards excitatory instead of inhibitory synapses as synaptosome fractions are generally enriched in excitatory synapses, which detach and reseal easier during the isolation process compared to inhibitory ones (10). In immunostaining of brain sections, there is no such difference. This methodical difference might also explain the dissimilarities between flow cytometry and immunostaining results, taking into account that NPs are exclusively located in the excitatory synapses (53) in contrast to C1q which likely tags inhibitory synapses as well (56).

### Neuronal Pentraxin 1 Is Predominantly Located in the Synaptic Plasma Membrane Fraction

**Figure 5:**
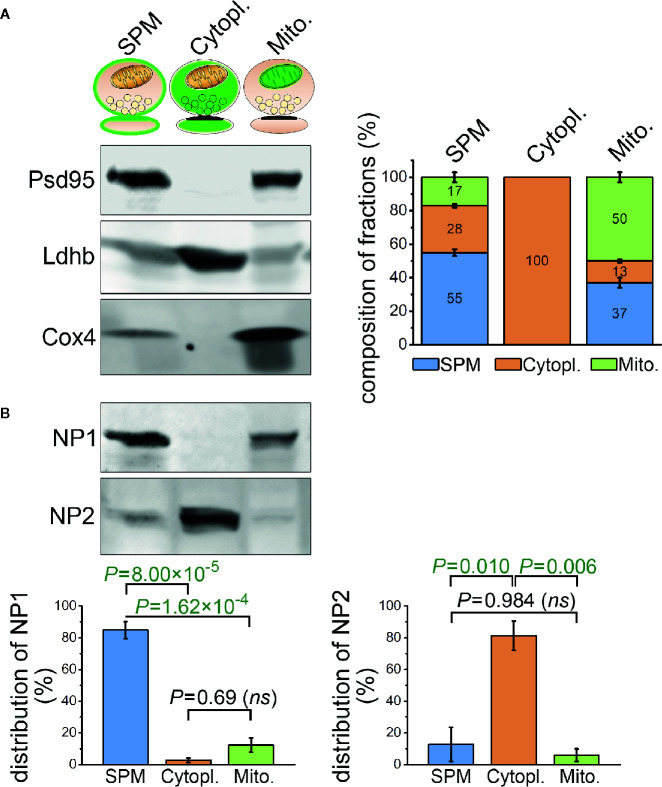
Differential sub-synaptic localization of NP1 and NP2. **(A)** Characterization of the synaptic plasma membrane (SPM), synaptic cytoplasm (Cytopl.), and synaptic mitochondria (*Mito.*) fractions by western blot technique. The representative western blot image (left) demonstrates the enrichment of postsynaptic density protein 95 (Psd95), L-lactate dehydrogenase B chain (Ldhb), and cytochrome c oxidase subunit 4 (Cox4) protein markers in their respective compartments, while the stacked bar graphs (right) depict the results of the quantification. **(B)** NP1 is predominantly located in the SPM fraction, while NP2 is enriched in the synaptic cytoplasm. Means ± S.E.M. are shown; *n* = 4 mice. Statistically significant differences were identified using one-way repeated measures ANOVA, followed by Bonferroni *post hoc* test.

In spite of the abundant colocalization between C1q and NP1 and to a lesser extent NP2 in brain sections, the question emerges, to what extent NPs are exposed on the synaptic surfaces enabling C1q-binding or present intracellularly in the synapse. Unfortunately, light microscopy of tissue sections cannot distinguish between intra- and extracellular protein localizations. Thus, we sought to complement microscopy data with a detailed assessment of the subcellular distribution of NPs by subcellular fractionation. Synaptosomes were fractionated to consecutively purify the synaptic cytoplasm, synaptic mitochondria, and synaptic plasma membrane using a previously described protocol (40). The purity of the fractions was tested with selective subcellular protein markers and their compositions were analyzed (**Figure 5A**) revealing the subcellular fraction-specific corrected levels of NPs. One-way ANOVA tests pointed out statistically significant differences for both NP1 (F(2,6) = 78.418, P = 0.00005) and NP2 (F(2,6) = 15.940, P = 0.00397) between their levels in the different compartments. We found that synaptic NP1 is primarily enriched in the synaptic plasma membrane fraction, while the majority of NP2 is localized in the synaptic cytoplasm (**Figure 5B**). Therefore, it is plausible that the abundant C1q-colocalized synaptic NP1 proteins observed in mouse brain sections are primarily located in the synaptic surface instead of the synaptic intracellular space, assuming their physical binding. On the other hand, the largest pool of synaptic NP2 is accumulated intracellularly implying that C1q-colocalized synaptic NP2 molecules observed by microscopy are, in fact, partly separated from extracellular C1q by the synaptic plasma membrane.

### The Majority of the Engulfed C1q-Tagged Synapses in Microglia Is NP1-Positive

**Figure 6:**
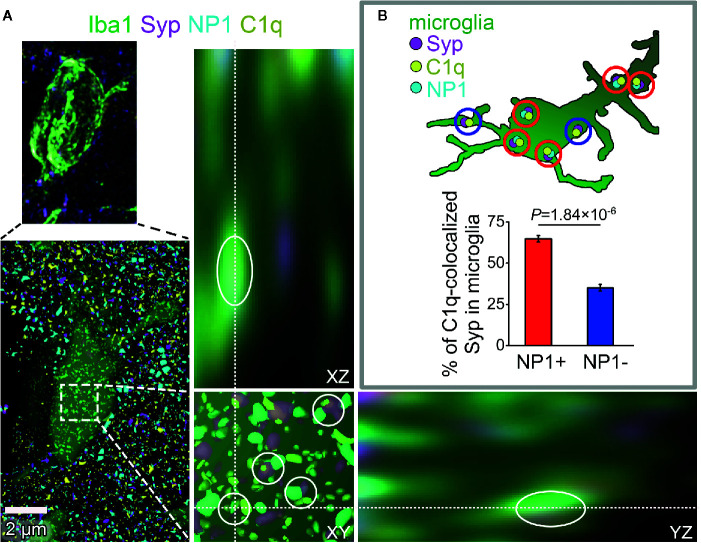
The presence of NP1 in microglial engulfed C1q-tagged synapses. **(A)** Microglia were reconstructed from confocal image stacks using Iba1-staining (green) and engulfed, C1q- and Syp-colocalized NP1 spots (yellow, magenta, and cyan, respectively) were identified on mouse brain sections (*white cycles*). Orthogonal views (XY, YZ) demonstrate that microglia completely surround one of the phagocytosed C1q-tagged synaptic material with NP1 content. **(B)** Image analyses revealed that 64.80 ± 1.97% of C1q-tagged microglial Syp proteins are NP1 positive as well. (Means ± SEM are shown; *n* = 16 images recorded from brain sections of three mice. Statistically significant difference between groups was identified using two-tailed Student *t*-test of paired samples.)

Our experiments have provided strong evidence using multiple methods for the direct interaction between C1q and NPs, with particular emphasis placed on NP1. Nevertheless, it remained unclear whether synaptic C1q–NP1/2 interactions influence microglial phagocytosis of synapses directly *via* the local complement system. Therefore, we carried out additional investigations to assess the levels of synaptic NPs in engulfed, C1q-tagged synaptic material inside the cytoplasm of the microglia. Similarly to our prior examinations, synaptic C1q and NPs in brain sections were initially identified by high-resolution confocal microscopy and image analyses. Then we reconstructed microglial processes based on immunostaining for the microglia marker Iba1. Finally, synaptic C1q and NP1/2 spots completely surrounded by microglia were assessed, and the ratio of C1q-tagged synapses with and without NP1/2 inside the microglia was evaluated. It has been demonstrated that both NPs and Syp derive mostly from neurons, lacking microglial expression [*e.g.*, the Brain RNA-Seq open-access database, https://www.brainrnaseq.org/ (57)], implying that Syp-colocalized NPs detected inside the microglia likely belong to phagocytosed but not yet digested synaptic material. In contrast, C1q-expression in the cerebral cortex is strictly limited to the microglia (58). Therefore, we have only taken into account those intracellular C1q spots within the microglia that showed strong colocalization with Syp indicating that they were secreted, synaptically deposited, and likely phagocytosed as part of the removed synapse by microglia. Our experiments demonstrated that the majority of C1q-tagged, microglia-surrounded synapses (~65%) show colocalization with NP1 as well (**Figure 6**), supporting the hypothesis that microglia predominantly eliminate in a complement-dependent manner those synapses that expose NP1 on their surfaces. On the contrary, NP2 was present in only ~28% of the microglia-surrounded C1q-tagged synapses (**Supplementary Datasheet 2**), suggesting that it is less involved in complement-mediated synaptic pruning. Taken together, our data support the hypothesis that the direct binding of C1q to synaptic NP1 is important for synapse elimination on the microglia–complement axis. Further studies are needed to prove this hypothesis and clarify the role of NP1 by investigating synaptic C1q and NP1 colocalization with phagocytic markers.

### Synaptic Presence of C4

**Figure 7:**
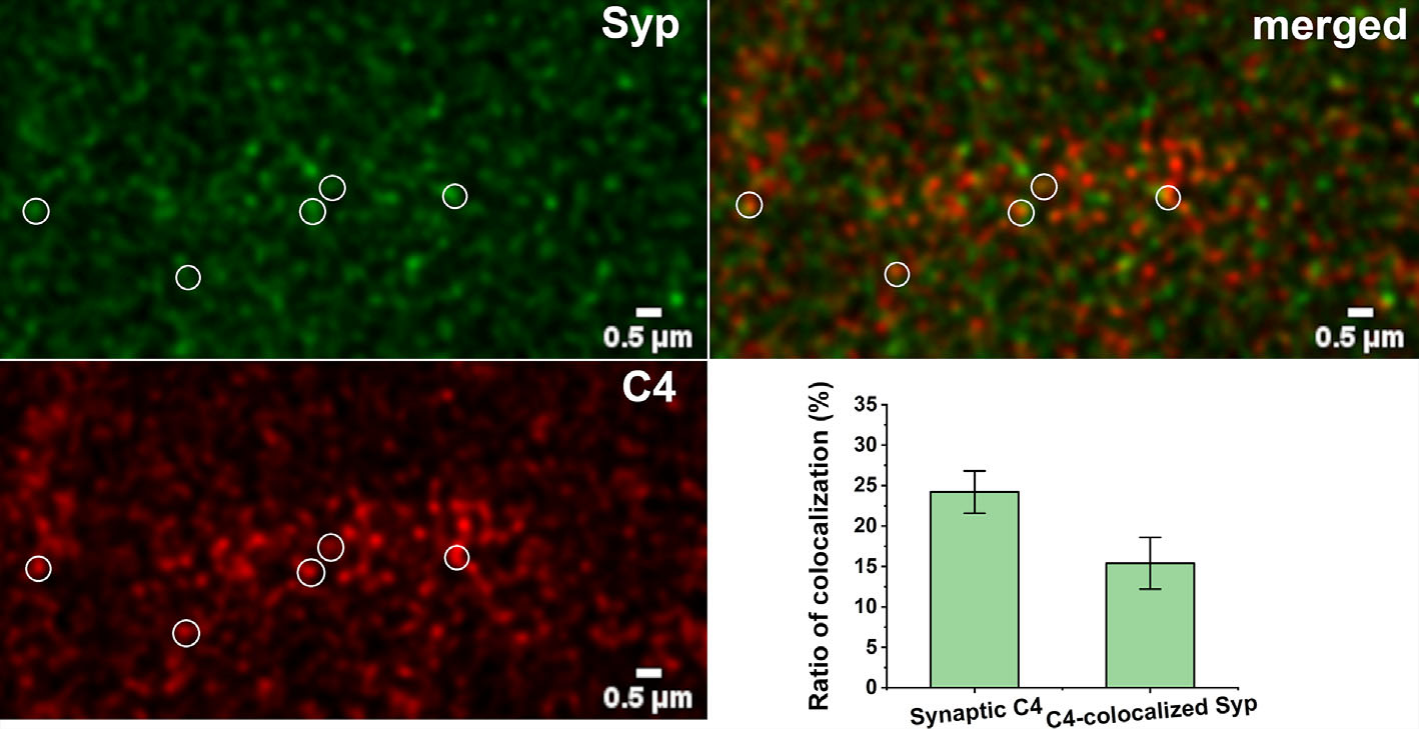
Synaptic presence of C4. High-resolution confocal microscopy images of double immunostained cerebral cortical sections were analyzed for colocalization of C4 and synaptophysin (Syp). 24.2 ± 2.6% of C4 showed synaptic localization. 15.4 ± 3.2% of synatophysin colocalized with C4. Some of the colocalizing spots are highlighted with white circles. Means ± SEM are shown; *n* = nine images recorded from sections of three mice. Scale bar = 0.5 µm.

There is an intriguing question if C1q action is independent from the complement cascade activation in our system. Beth Stevens and Steven McCarrol showed that gene variants that boosted C4 expression the most were associated with higher schizophrenia risk, suggesting that high levels of the protein could promote excess pruning (59). On the other hand, Stephan and co-workers showed that the level of C1q in the brain is significantly increased with normal aging, more than that of the other components, such as C3 (56). This would suggest that C1q might have an independent function in the brain. We investigated the presence and synaptic localization of C4 *via* immunostaining of mouse cortical brain sections combined with pseudo-super-resolution confocal microscopy and automated image analysis. We clearly observed the presence of C4 in the brain sections of the adult mice. The results showed that 24% of C4 is synaptically localized (**Figure 7**). This high-level presence of synaptic-localized C4 suggests that the classical complement pathway might play a role in the synaptic functions of the complement, and C1q is not a single player. To discover the exact mechanism and pathways will require more investigations.

## Discussion

Generally, synaptic connections are not rigid but undergo constant remodeling in response to external and internal stimuli. Proper operation of this structural synaptic plasticity relies on the equilibrium between synaptogenesis and synaptic pruning even in the adult brain, which is the target of our investigations. However, its distortion, such as extensive synapse loss in neurodegenerative conditions, can lead to severe functional disturbances. Several molecular partners are known to regulate this balance (60); however, we are still far from the detailed mechanistic understanding of synaptogenesis and selective synapse removal. Recently, complement proteins came into the focus of interest because accumulating evidence pointed out their role in synaptic pruning. Among them, probably C1q is responsible for the selectivity in synapse recognition (20), but the exact role of the complement cascade in the CNS in this mechanism is still elusive. Although little is known about the synaptic interaction partners of C1q in the CNS, NPs appear to be potential targets as suggested by their structural homology to peripheral C1q binding partners of the pentraxin protein family (20–22) and their previously described role in synaptic refinement in the developing visual system (23). Our present study systematically addressed this hypothesis and revealed NP1 and NP2 as two novel synaptic C1q binding partners in the adult brain. Moreover, we hypothesize that NP1 is linked to the selective elimination of C1q-tagged synapses by the microglia. We have to note that our results are valid for synaptic pruning in the adult mice and would need further investigations to verify the applicability for the young developing brain.

Our investigations incorporated both *in vitro* and *in vivo* experiments to unambiguously prove the physical interaction of NPs with C1q and to reveal its potential synaptic importance. To overcome the inherent limitations and obstacles of each technique, we strove for employing mutually complementary methods. Initially, we addressed whether NPs mimic the C1q-binding abilities of PTX3 surmised by their remarkable structural homology. Our *in vitro* experiments verified the direct binding of isolated NPs to C1q, which was strong enough to elicit downstream complement activation (**Figure 1**). This result is in agreement with the well-known binding of immobilized PTX3 to C1q in the periphery leading to the activation of the classical complement pathway on the surfaces of apoptotic cells (21, 61). Nevertheless, it was previously reported that pre-incubation of PTX3 with C1q in the fluid-phase acts oppositely and causes inhibition of complement activation (21). Contrary to this behavior, our microtiter plate binding assays demonstrated that NP1/2–C1q binding results complement activation in both the fluid and immobilized phases (**Figure 1**). As PTX3 mediates complement activation in the periphery, NPs might be capable of influencing the possible activation of the classical pathway in the brain, which idea is strengthened by our complement activation results.

Currently, the *in vivo* function of C1q–NPs binding in the extracellular milieu without being immobilized to the cell surface is unknown. Theoretically, their extracellular interaction can either serve as a means to deplete deposition of the “eat-me” signal complement components onto the cell surface or can aid complement accumulation *via* their tethering by membrane-bound NP receptors. Therefore, further studies are warranted to verify the assembly of the C1q–NP1/2 complex in the extracellular space *in vivo* and clarify its possible role.

PTX3 interacts with the classical and lectin pathway regulator C4BP and the alternative pathway regulator Factor H preventing exaggerated complement activation that would otherwise lead to inflammation and host tissue damage (52, 62, 63). According to our results, NPs can bind to both C4BP and Factor H (**Supplementary Datasheet 2**). The phenomenon may stem from the structural homology of pentraxins, and in the future, it may be worth exploring whether these connections have a significance in the brain.

Our data suggest that C1q–NP1/2 complex is present in the synapses. Importantly, the great extent of their colocalization was demonstrated by co-immunoprecipitation, flow cytometry, and high-resolution confocal microscopy investigations, ruling out the possibility that the formation of their synaptic complex is merely artificial (**Figures 3**, **4**). The techniques we used allowed for specifically studying synaptically bound C1q–NP1/2 complexes, and the obtained data indicate that the C1q–NP1 complex is present on the synaptic surface. Thus, the question may arise where the synaptic C1q–NP1 complex could be formed within the synapse. The classically known C1q, built-up by 18 subunits is an outstandingly large protein with a size that almost exceeds the average size of the synaptic cleft (~20 to 40 nm) (64). Taking into account this size limitation, the pool of secreted C1q that mediates selective pruning of synapses is likely attached to perisynaptic regions instead of direct binding into the synaptic junction. Previous data showing primarily perisynaptic C1q binding *via* immunogold electron microscopy examinations also supports this notion (56). Considering that NPs within the synaptic junction are well-known binding partners of AMPA receptor (AMPAR) subunits, we hypothesize that C1q binds to a pool of perisynaptic and therefore possibly not AMPAR-bound NPs. In agreement with the literature, we confirmed the presence of NPs in the synapses *via* immunocytochemistry, flow cytometry, and immunohistochemistry. However, conspicuously, NPs were not uniformly present in all synapses, which might imply that, despite the fact that NPs are considered common synaptic proteins, functional differences between synapses may cause a heterogeneous distribution of NPs. Regarding synaptic functional differences, NP2 is an immediate early gene translated by neuronal activation (29), while the expression of NP1 is increased under reduced activity (33). Moreover, NP1 and NP2 are also known to form heterocomplexes (25). Thus, the apparent contradiction between their expression pattern and assembly needs to be clarified.

Our current results repeatedly demonstrated the high but certainly not complete colocalization between C1q and NPs (**Figures 3**, **4**). This observation implies the critical role of yet unknown rules that determine selective recognition of certain synaptic NPs by C1q. Although synaptic NPs are capable of complement binding (**Figure 1**), they probably have independent functions in parallel that explains their uneven C1q-binding pattern. As already described, NPs are involved in AMPAR clustering at the excitatory synapses, and by this mechanism, they can regulate excitatory synaptogenesis, functional synaptic plasticity, and synapse formation (24–27). Moreover, NP1 plays a role in neuronal cell death under hypoxic–ischemic conditions (36) and mediates the accumulation of the pro-apoptotic BAX protein on the mitochondrion’s surface (35). On a theoretical basis, several explanations could be offered to clarify the partial recognition of NPs by the synaptic C1q. First, the ratio of NP1 and NP2 within their heterooligomeric complex can vary, influencing, *e.g*., their AMPAR binding characteristics (25). A particular subunit composition of the perisynaptic NP complex that does not favor AMPAR binding might, instead, facilitate synaptic C1q deposition. Second, posttranslational modifications of NPs could also influence their C1q-binding characteristics. It has been reported that NP1 is subjected to glycosylation (65), and we previously assumed its phosphorylation as well (18) in agreement with prior screening data [PhosphoSitePlus database (66)]. Taking into account that sialic acid in the neuronal glycocalyx prevents C1q deposition (67), and PTX3 can possess sialic acid residues modulating its C1q-binding capabilities (68), the presence of a unique NP glycosylation pattern with or without sialic acid might determine the strength of the NP–C1q interaction on the synaptic surface as well.

Our subcellular fractionation experiment demonstrated the differential localization of NPs within the synapse (**Figure 5**). Consistent with its AMPAR clustering function, NP1 is enriched in the synaptic plasma membrane with detectable levels in the synaptic mitochondrial fraction as well. Its mitochondrial identification is in accordance with prior data showing the role of NP1 in the intrinsic mitochondrial pathway of apoptosis (33, 69). In contrast, the majority of NP2 was identified in the synaptic cytoplasm fraction and, to a lesser extent, in the SPM. Although NP2 is primarily known as a synaptic protein, which binds AMPARs, our results demonstrate their prominent intracellular accumulation as well. Considering that NP2 is an immediate early gene (32), it can be hypothesized that the presence of its cytoplasmic pool is the result of its persistent activity-dependent translation without reaching secretion.

In addition to describing the C1q–NP binding, we investigated the biological role of this interaction in synapse elimination. Our results raises the probability that C1q-tagged, primarily NP1-positive synapses are engulfed by microglia (**Figure 6**). Thus, NP1 could facilitate the binding of C1q to synapses to be removed. On the other hand, NP2 alone is unlikely to serve as an “eat-me” signal for complement components in the course of synapse phagocytosis (**Supplementary Datasheet 2**). Although the exact subunit composition of the NP assembly that is involved in synapse elimination remains elusive, our results imply that the complex consists predominantly of NP1 instead of NP2. It is already known that C1q is deposited onto synapses with reduced activity (11) and where apoptotic-like mechanisms are triggered (18). In line with these data, the initiation of local synaptic apoptotic mechanisms is preceded by a decrease in synaptic transmission (70). It was assumed that in a very similar manner to C1q, NPs are also involved in the elimination of low-activity synapses during synaptic pruning at early ontogenesis (23). Moreover, members of the pentraxin family at the periphery play key role in the elimination of apoptotic cells *via* phagocytosis, which process is regulated *via* the interaction between pentraxins and complement components, such as C1q and complement pathway inhibitors (22, 52, 61, 63, 71). Combining these prior data and our current results, we propose that NP1 aids the removal of apoptotic synapses with reduced synaptic strength *via* the complement–microglia pathway. The presence of synaptic C4 suggests that activation of the classical pathway might occur in this adult mouse model which is in line with previous reports on the role of C4 in synapse elimination (59). We propose that preventing excessive synaptic exposition of the C1q-binding form of NP1 or blocking the C1q–NP1 interaction could be beneficial to arrest increased complement-dependent synapse loss, which is a major component in the pathogenesis of AD and other neurodegenerative diseases (13).

Finally, accumulating evidence implies that NPs might be promising cerebrospinal fluid (CSF) and blood biomarkers of early AD (72–74). In addition, amyloid-*β*-induced increase in NP1 expression has been linked to neuronal toxicity in AD (37). In contrast, NP2 is downregulated in *post mortem* human AD brain specimens, which correlates with the reduction of the AMPAR subunit GluA4. Moreover, reduced NP2 levels were identified in the CSF of AD patients, and NP2 amounts showed a robust positive correlation with cognitive performance and hippocampal volume (74). Therefore, both NP1 and NP2 can be regarded as important CSF or blood biomarkers of AD, but the background of their different levels will have to be clarified in future studies.

In summary, our results clearly demonstrate the interaction between C1q and NPs on the synapse using a wide array of *in vitro* and *in vivo* examinations. Moreover, we hypothesize the preferential engulfment of NP1-containing synapses *via* the complement-microglia axis.

## Data Availability Statement

The raw data supporting the conclusions of this article will be made available by the authors, without undue reservation.

## Ethics Statement

The animal study was reviewed and approved by the Ethics Committee for animal studies of Eötvös Loránd University, Budapest, Hungary. The care and treatment of all animals conformed to guidelines approved by Council Directive 86/609/EEC and the Hungarian Act of Animal Care and Experimentation (1998, XXVIII) as well as with local regulations for the care and use of animals for research. All efforts were taken to minimize the animals’ pain and suffering and to reduce the number of animals used.

## Author Contributions

RK, HV, ÉB, VT, DM, JuK, GT, FF, EG, ÁC, and BG performed experiments. RK, VT, ÉH-G, BG, and JóK analyzed results. KM, LH, MJ, GJ, KK, BG, and JóK designed the work and supervised experiments. RK, KK, BG, and JóK wrote the manuscript. All authors contributed to the article and approved the submitted version.

## Funding

This study was supported by the National Research, Development and Innovation Office of Hungary (grants KTIA_NAP_13-2-2014-0017 and 2017-1.2.1-NKP-2017-00002, FIEK_16-1-2016-0005, VEKOP-2.3.3-15-2016-00007, GINOP-2.3.2-15-2016-00020, and K128123) and the Ministry of Human Capacities (grant NTP-NFTÖ-19-B-0057 and *Institutional Excellence Program, ELTE ID: D11206*), and the EU’s Horizon 2020 research and innovation program under grant agreement No. 739593.

## Conflict of Interest

The authors declare that the research was conducted in the absence of any commercial or financial relationships that could be construed as a potential conflict of interest.
