# Supplementary material for: Identification of Neuronal Pentraxins as Synaptic Binding Partners of C1q and the Involvement of NP1 in Synaptic Pruning in Adult Mice
Source: Front Immunol. 2021 Feb 8;11:599771. doi: 10.3389/fimmu.2020.599771 (PMC7897678; doi:10.3389/fimmu.2020.599771)
Supplement: Supplementary file 2 [file DataSheet_2.pdf]

Supplementary Material

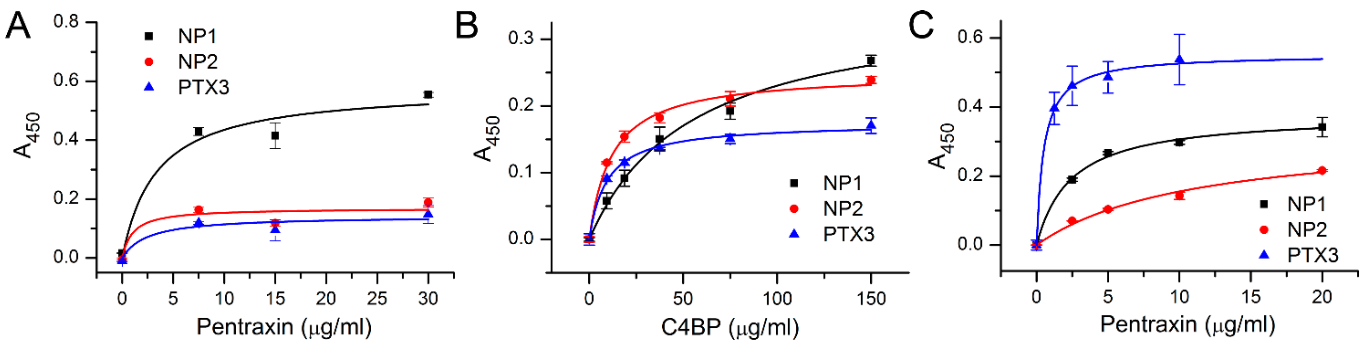

Supplementary Figure 1. Binding of neuronal pentraxins to C1q and inhibitors of the classical and alternative complement pathways. (**A**) ELISA assay by coating C1q to the plate and applying NP1, NP2, and PTX3 in the fluid phase (opposite arrangement to Figure 1a). Representative measurements are shown. (**B**) We investigated whether, analogously to PTX3, NPs bind to C4BP, the main soluble inhibitor of the classical pathway. NPs were coated in this experiment. Means ± SEM; n = 4. (**C**) Factor-H, the main soluble inhibitor of the alternative pathway was coated. NPs in the fluid phase showed dose-dependent binding in this representative experiment. Means ± SEM; n = 3. Plots were fitted with hyperbolic function.

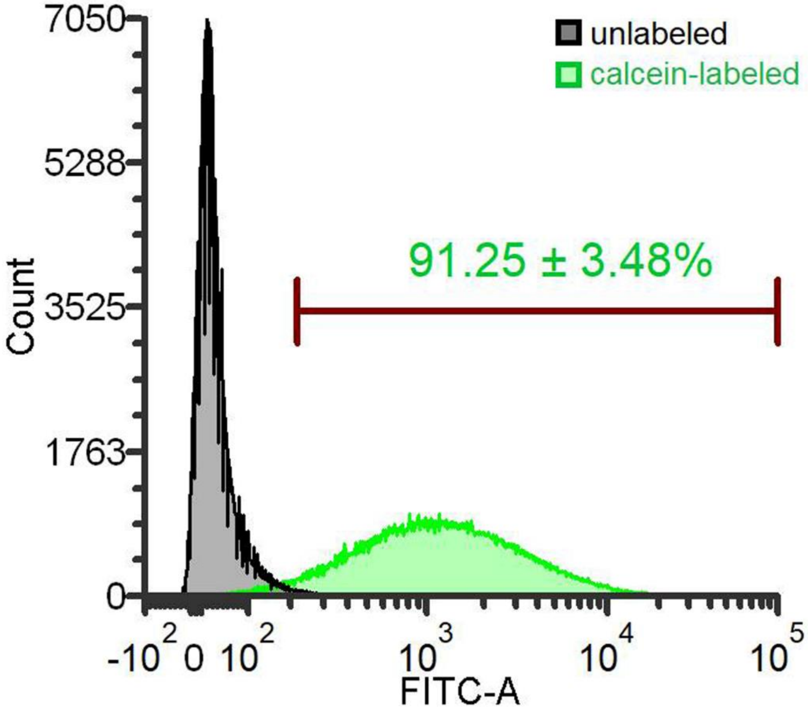

Supplementary Figure 2. Calcein-AM labeling was applied to test the viability of synaptosomes and validate the exclusive binding of antibodies to the synaptic surface. Calcein labeling was done separately and the calcein-labeled samples went through the same procedure as the antibody-labeled samples.

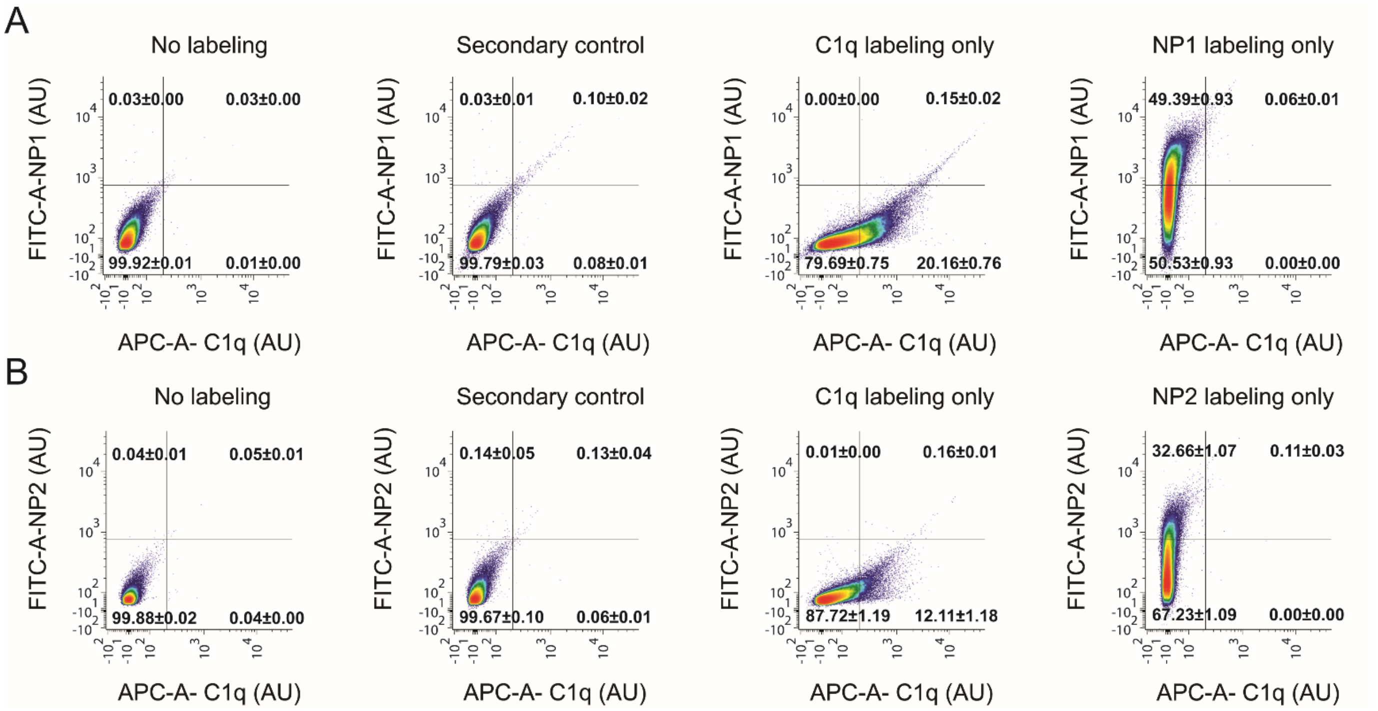

Supplementary Figure 3. FACS control measurements showing non-labeled, only secondary antibody-labeled, only primary antibody-labeled samples. These controls demonstrate that there is no spillover between the channels APC and FITC.

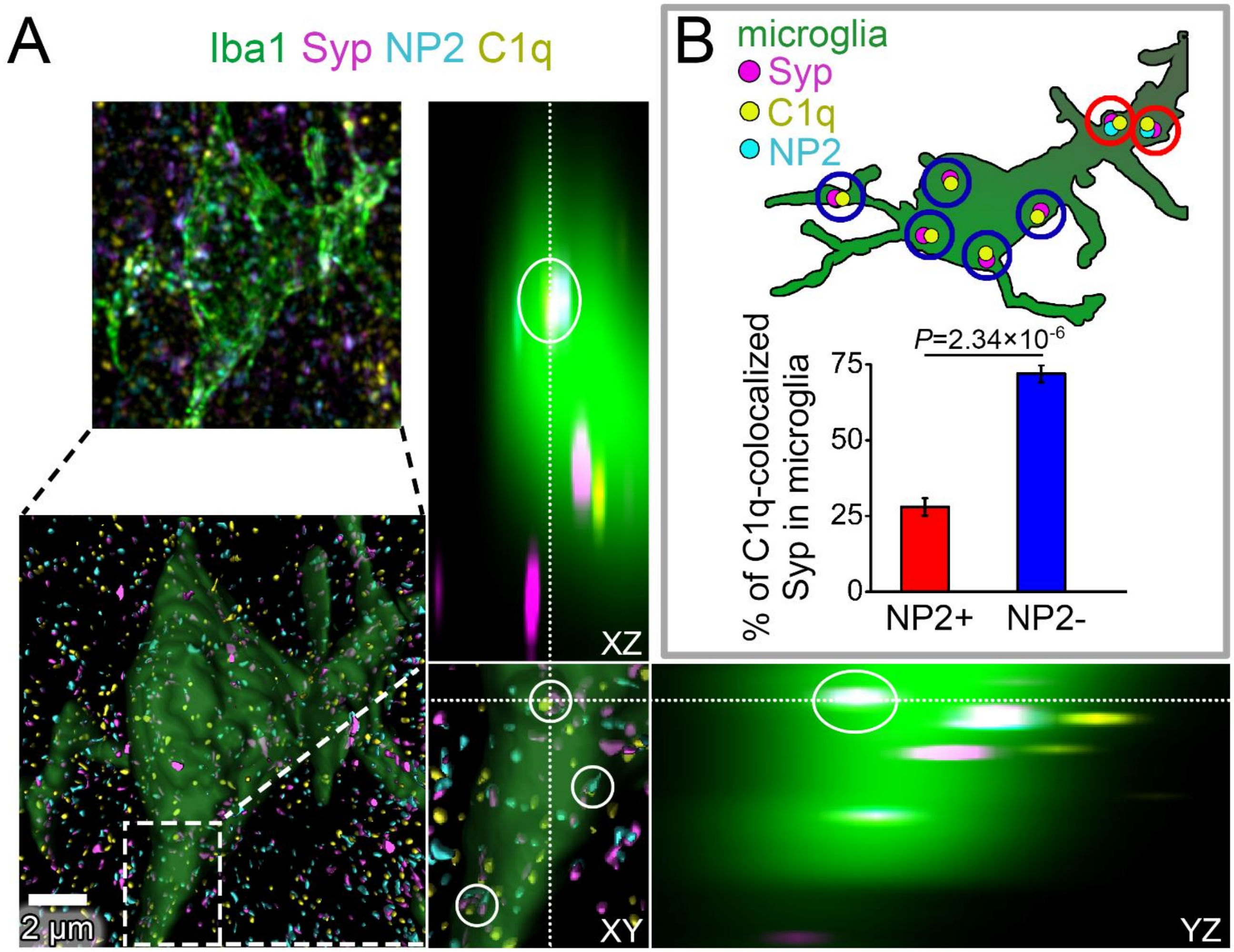

Supplementary Figure 4. Microglial engulfed C1q-tagged synapses are mostly NP2-negative. (**A**) Microglia were reconstructed using Iba1-staining and engulfed, C1q- and Syp-colocalized NP2 spots were identified on mouse brain sections (*white circles*). Orthogonal views demonstrate that microglia completely surround one of the phagocytosed C1q-tagged synaptic material with NP2 content. (**B**) Image analyses revealed that only 28.01 ± 2.88% of C1q-tagged microglial Syp proteins are NP2-positive as well, while the majority of them lacks synaptic NP2. Means ± SEM are shown; n = 16 images recorded from brain sections of 3 mice. Statistically significant difference between groups was identified using two-tailed Student t-test of paired samples.
